# Neoplastic Meningitis from Solid Tumors: A Prospective Clinical Study in Lombardia and a Literature Review on Therapeutic Approaches

**DOI:** 10.1155/2013/147325

**Published:** 2013-01-16

**Authors:** A. Silvani, M. Caroli, P. Gaviani, V. Fetoni, R. Merli, M. Riva, M. De Rossi, F. Imbesi, A. Salmaggi

**Affiliations:** ^1^Fondazione I.R.C.C.S. Istituto Neurologico C. Besta, Milano, Italy; ^2^Clinica Neurochirurgica, Ospedale Policlinico, Milano, Italy; ^3^Ospedale Melegnano, Milano, Italy; ^4^Ospedali Riuniti Bergamo, Milano, Italy; ^5^Ospedale di Lodi, Lodi, Italy; ^6^Ospedale Niguarda, Milano, Italy; ^7^Ospedale Lecco, Lombardy, Italy

## Abstract

Neoplastic dissemination to the leptomeninges is an increasingly common occurrence in patients with both haematological and solid tumors arising outside the central nervous system. Both refinement of diagnostic techniques (Magnetic resonance imaging) and increased survival in patients treated with targeted therapies for systemic tumors account for this increased frequency. Cerebrospinal fluid cytological analysis and MRI confirm clinical diagnosis based on multifocal central nervous system signs/symptoms in a patient with known malignancy. Overall survival in patients with leptomeningeal neoplastic dissemination from solid tumors is short, rarely exceeding 3-4 months. However, selected patients may benefit from aggressive therapies, Apart from symptomatic treatment, intrathecal chemotherapy is used, with both free (methotrexate, Thiotepa, AraC) and liposomal antitumor agents (liposomal AraC). Palliative radiotherapy is indicated only in cases of symptomatic bulky disease, surgery is limited to positioning of Ommaya recervoirs or C5F shunting. We report clinical data on a cohort of 26 prospectively followed patients with neoplastic leptomeningitis followed in Lombardia, Italy, in 2011. Prognostic factors and pattern of care are reported.

## 1. Introduction

Neoplastic meningitis is due to dissemination of malignant cells to the leptomeninges and the subarachnoid space. It occurs in 10–15% of haemolymphoproliferative malignancies and in 5–10% of solid cancers [1].

It more frequently represents late complication of long-standing neoplastic disease, but in 10–15% of patients may be the first-ever manifestation of otherwise occult cancer [1].

The pathways for tumor dissemination to the leptomeninges and subarachnoid space include haematogenous route, perineural blood/lymphatic vessels, and direct infiltration from contiguous sites (for instance, dural and/or bone metastases close to the brain and spinal cord/root surface).

Not only extra-CNS tumors, but also tumors arising within the CNS (among which gliomas, ependymomas, medulloblastomas, and germinomas) display relapses and/or multifocal presentations with distant foci and a supposedly intra-CSF pathway of dissemination of neoplastic cells.

Guidelines for effective treatment of neoplastic meningitis are lacking, due to the low levels of evidence, which is mostly present for haemolymphoproliferative disease.

In meningeal dissemination from solid extra-CNS tumors, and more so in distant spread of primitive CNS tumors, there is a lack of uniform approach due to a number of factors: among these, the belief of oncologists that neoplastic meningitis invariably implies a dismal prognosis in the short-term has limited patient recruitment in clinical trials.

Although this assumption holds true in a high number of cases, it does not apply to the totality of patients, however.

This consideration, together with the more widespread availability of MRI facilities in neurooncological diagnosis and with the progress in survival in extra-CNS cancers achieved by chemotherapy and molecularly targeted therapies [2], increases the need for accurate diagnosis of neoplastic meningitis, as a prerequisite for accurate validation of prognostic factors and for enrollment of patients in clinical trials.

## 2. Diagnosis of Neoplastic Meningitis

The clinical signs and symptoms of neoplastic meningitis are classically subdivided in those pointing to cerebral, cranial nerve, or spinal cord/roots involvement. Typically, in a high proportion of patients symptoms are present suggesting simultaneous involvement of both cerebral and spinal levels, but some patients present with isolated deficits (for instance, an isolated cranial nerve defect).

Cerebral signs and symptoms may either be localized (as in the case of focal seizures) or suggestive of a widespread brain dysfunction (for instance, drowsiness in hydrocephalus or encephalopathic features in diffuse sulcal enhancement), or be even more unspecific, such as headache.

The literature reports that the presence of signs at the neurological examination is more frequent as compared to the reporting of symptoms by the patients during history collection.

Neoplastic meningitis not infrequently coexists with intraparenchymal or dural metastases, especially in the case of breast cancer and leukemia/lymphoma.

The diagnosis of neoplastic meningitis is straightforward in the majority of cases, but a number of cases may pose diagnostic challenges.

This happens more frequently when the gold standard for diagnosis (i.e., CSF cytology) does not yield unequivocally positive results. This may be the case—according to the literature—in a proportion of patients ranging from 20 to 50–60%; reasons for this include too little volume of CSF analyzed, distance of the CSF sampling site from the bulk of leptomeningeal disease, and delay in CSF processing and analysis [3, 4]. The diagnostic yield of CSF cytology increase significantly from the first to the second lumbar puncture, to rise only negligibly thereafter [5].

In such cases, CSF analysis may yield negative results for malignant cells, yet display other abnormal features (however, less specific), such as increase in total proteins and reduced glucose levels, as well as moderate reactive pleocytosis.

Such CSF pattern may pose serious difficulties in differential diagnosis with CNS infections, which may mimic the neuroradiological picture of NM and are not unexpected in heavily treated cancer patients (for instance, chronic fungal and/or mycobacterial meningitis).

Some reports have stressed that the closer the CSF sampling to the site of disease, the higher the percentage of positivity for CSf malignant cells; ventricular CSF or lumbar CSF may thus provide different information as far as cytology is concerned.

In exceptional cases, leptomeningeal biopsy is deemed necessary.

In neoplastic meningitis from heamatological malignancies, CSF cytofluorimeter analysis has been reported to be more often diagnostic as compared to standard cytomorphological analysis [6, 7].

As far as the role of MRI is concerned, the features of leptomeningeal dissemination include both indirect and direct evidence of neoplastic cell CSF seeding. Among the former, hydrocephalus is not rare, due mostly to alterations in the CSF flow and particularly in CSF reabsorption at the skull vault. Direct evidence of neoplastic dissemination includes linear or nodular enhancement at leptomeningeal/ependymal level.

More subtle signs of alterations in the CSF dynamics include exclusion of part of cerebral sulci, with limited volumes with increased protein content.

## 3. Management of Neoplastic Meningitis

The role of surgery is limited to resection of symptomatic, bulky disease, and/or biopsy in order to achieve diagnosis in selected cases; in some patients, positioning of an Ommaya recervoir may allow intraventricular chemotherapy without the need for repeated lumbar punctures, but the dynamics of CSF flow need to be carefully assessed in order to possibly achieve tumoricidal drug concentrations in the sites of disease. Ventriculoperitoneal shunting procedures to relieve symptomatic hydrocephalus carry a risk for the development of neoplastic dissemination to the peritoneum and are often complicated by shunt dysfunction/occlusion.

Intrathecal chemotherapy should preferably be delivered in patients with good PS (see below), with limited extra-CNS disease and with linear contrast enhancement at MRI (the penetration of drugs within bulky disease areas is limited to 2-3 mm).

The NCCN 2012 Guidelines for diagnosis and management of CNS tumors include brain and spine MRI as well as CSF examination in the workup of patients with suspected leptomeningeal tumor dissemination. According to these guidelines, either positivity of CSF cytology alone or positive radiologic findings with supportive clinical findings or else signs and symptoms with suggestive CSF in a patient known to have a malignancy may be sufficient for diagnosis.

After diagnosis, patients are stratified in either poor risk (low KPS, multiple, serious, major neurologic deficits, extensive systemic disease with few treatment options, bulky CNS disease, and encephalopathy), or else good risk (high KPS, no major neurologic deficits, minimal systemic disease, and reasonable systemic treatment options).

In the former group, only fractionated external beam RT is considered to symptomatic sites, and palliative care is the standard. An exception is possible in patients with highly chemosensitive tumors such as lymphoma and SCLC.

On the other hand, in good risk patients both radiotherapy to bulky disease or symptomatic sites may be delivered and intrathecal chemotherapy is a worthwhile option.

Of note, assessment of CSF flow is strongly recommended before initiating intrathecal chemotherapy. This assessment is more frequently performed in northern America, while it is less a frequent practice in Europe.

With normal CSF flow, either craniospinal irradiation—in the case of breast cancer or lymphoma—or high dose methotrexate i.v in the case of breast cancer or lymphoma or intrathecal chemotherapy with methotrexate or AraC or liposomal AraC are the treatment of choice.

Unless an Ommaya recervoir is positioned by the neurosurgeon, repeated intrathecal administration of antineoplastic drugs is usually performed via lumbar punctures. With methotrexate, twice weekly administrations are performed during the induction phase, due to the short half life of the drug in the CSF.

Analogous schedules are needed with nonliposomal cytarabine, whereas a pegylated formulation of cytarabine allows sustained tumoricidal concentrations in the CSF which make once every 2 weeks treatment possible. The development of cytarabine encapsulated in multivesicular liposomes has led to detection of CSF concentrations of more than 0.1 *μ*G/mL persisting at 14 days.

In this technology, microscopic particles made of aqueous chambers separated from each other by bilayer lipid membranes (with synthetic analogs of natural lipids), deliver gradually the incorporated drug, with subsequent metabolization of the membrane remnants via normal pathways. Cytarabine, a highly hydrophyilic compound, is an ideal molecule for this approach [8].

The achievement of tumoricidal concentrations of cytarabine in the CSF is of crucial importance since cytarabine is a phase-specific drug affecting only cells in the S phase. In the CSF, very little activity of the inactivating enzyme cytidine deaminase enables cytarabine to persist in its biologically active form for longer time as compared to systemic delivery [9].

Only few randomized trials have been conducted on the effectiveness and toxicity of intrathecal chemotherapy in neoplastic meningitis (reviewed in [10]).

In the 1999 published trial by Glantz et al. on neoplastic meningitis from solid tumors [11], intrathecal methotrexate was compared to liposomal cytarabine in 61 patients. After the induction phase, a slight increase in the frequency of patients attaining a response in the liposomal AraC group (26% versus 20%) was seen. Overall, median survival reached 73 days in the latter group and 105 in the former, with a nonsignificant advantage. The only parameter displaying a definite benefit in the liposomal AraC group was the time to neurological progression, which was of 58 versus 30 days with a statistically significant difference. It remains to be seen whether this statistically significant improvement translates into a clinically meaningful effect, but in this respect the studies conducted so far lack detailed quality of life data and this makes conclusions difficult.

Also the 2006 trial by Shapiro and colleagues provides data pointing to a nonsignificantly different effect of liposomal AraC versus methotrexate in 103 patients with neoplastic meningitis froms solid tumors [12].

In the other 1999 paper by Glantz et al. [13], liposomal AraC was compared to AraC in the treatment of neoplastic meningitis in a low number (28) of patients with lymphomatous meningitis. This trial showed an increase in time to tumor progression, in survival time and in response rate in the liposomal AraC treated subgroup.

Other nonrandomized studies have been performed [14, 15] investigating the effectiveness and side effects of liposomal cytarabine in neoplastic meningitis. Overall, a fair tolerability profile has emerged. The frequent occurrence of chemical meningitis may be prevented by concomitant steroid treatment. 

The main reason for continuing use of liposomal AraC in these patients—apart from the lack of a consolidated and effective standard of care—is the need for less frequent lumbar punctures in often severely ill patients. However, the levels of evidence in favour of this approach are weak. A recent determination of EMA has temporarily suggested to consider alternative therapies to liposomal AraC after an inspection to the production site of the drug in California; treating physicians are waiting for a solution of this possibily temporary problem. 

Other widely adopted intrathecal treatments apart from liposomal AraC include methotrexate and thiotepa.

Preliminary experiences show the feasibility of associating rituximab with liposomal cytarabine in patients with recurrent neoplastic meningitis [16]. Also systemic bevacizumab may be effective in some cases on neoplastic meningitis [17], in combination with other systemic chemotherapeutic agents.

Some effect has been reported for systemic treatment with systemic gefitinib or erlotinib in NSCLC with neoplastic meningitis, and with sorafenib in renal cancer, whereas the role of trastuzumab in breast cancer with neoplastic meningitis is still debatable (reviewed in [18]).

## 4. Prospective Collection of Newly Diagnosed Neoplastic Meningitis Cases from Solid Tumors in Lombardia

In 2011 a prospective collection of patients diagnosed with neoplastic meningitis from solid tumors was started in a number of Centers in Lombardia. The aim of this study is to assess the pattern of care in this often underdiagnosed and undertreated condition. Previous work from an analogous initiative in Piedmont [19] supports the concept that a higher index of suspect for diagnosis may lead to earlier diagnosis of this condition. Increase in frequency of neoplastic meningitis may indeed be a consequence of survival increase in a number of systemic malignancies thanks to advances in targeted therapies, as well as of more widespread use of MRI in the followup of these patients.

In 12 months, 26 patients with neoplastic meningitis from solid extra-CNS tumors have been diagnosed. Their clinical features are reported in Tables 1 and 2.

Cerebrospinal fluid analysis was performed in 22 out of 26 patients, yielding the following results: in 18/22 patients, CSF analysis revealed malignant cells. Mean values of CSF total protein were 152 mg% (normal values 10–45 mg%), whereas mean CSF glucose was 51.5 mg/dL (normal values 40–80 mg/dL for normal glycemic levels). Lower than normal glucose levels were only seen in 3 patients out of 22.

As reported in Table 3, 11 out of the 26 patients were treated by intrathecal liposomal AraC and 2 by systemic chemotherapy.

In this cohort, no patient was treated by radiotherapy after diagnosis of neoplastic meningitis.

**Figure 1:**
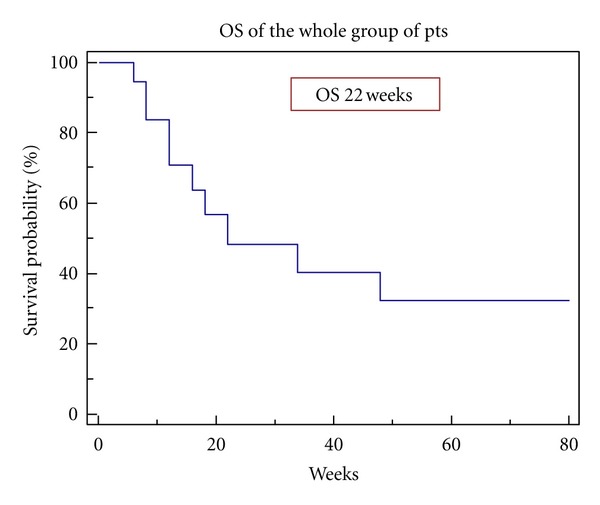


Figure 1 reports overall survival in the entire cohort. This attained a median value of 22 weeks, in line with data from the literature.

Assessment of possible prognostic factors showed that at univariate analysis, higher performance status, primary histology (breast versus others), less elevated CSF protein, and linear contrast enhancement at MRI versus nodular disease, as well as intrathecl chemotherapy versus no intrathecal chemotherapy were associated with more prolonged survival.

However, probably due to the low number of patients, no statistically significant differences were detected in subgroups at multivariate analysis.

**Figure 2:**
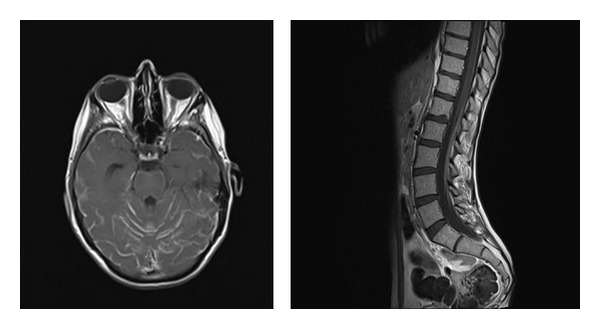
Postcontrast T1-weighted MRI images of diffuse enhancement in cerebral sulci and linear enhancement surrounding the dorsolumbar spinal cord and the lumbosacral roots in a 28-yr-old female with breast cancer.

In Figure 2 the MRI images of a young female affected by neoplastic meningitis from breast cancer are reported; this 28-yr-old woman had a 2-year history of ductal carcinoma Her2-, hormone receptor-negative with positive lymphnodes at diagnosis. She had been treated with systemic chemotherapy, surgery, second-line chemotherapy associated with antiangiogenic therapy for relapse, and with RT on lymhnodes. 18 months after diagnosis, she developed fever and headache, with subsequent rapid development of confusion, cognitive deterioration, behavior abnormalities, and progression to stupor. On neurological examination at admission, the patients was responsive but not oriented in space and time, with signs of meningeal irritation. She could not walk, the sitting position was maintained with difficulty. Cerebrospinal fluid analysis disclosed 90 cells (of which 85 malignant cells, cytokeratin-positive), with negative cultures, extremely low glucose levels (4 mg%), and slightly increased total proteins (64 mg%). Due to the very poor conditions, only palliative care was chosen for this patient, who died 4 weeks after diagnosis.

**Figure 3:**
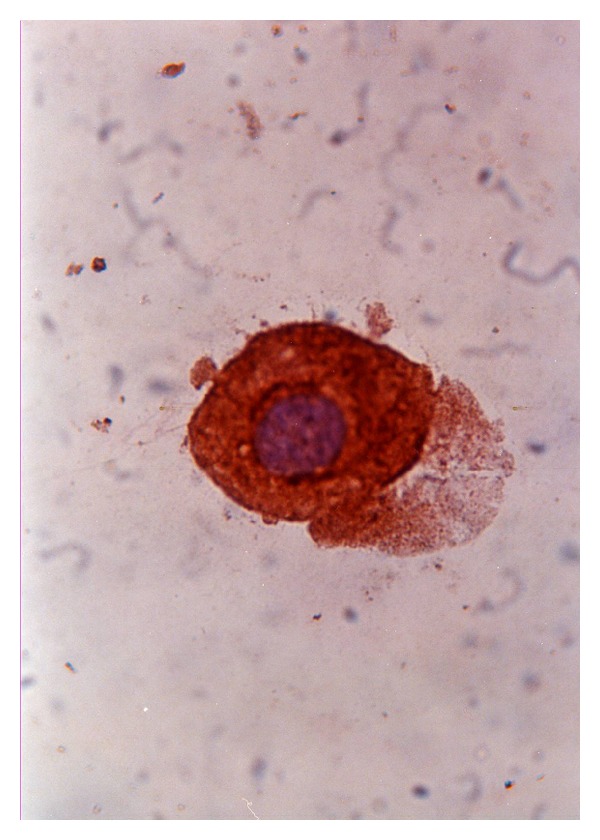
CSF cytology with stain with peroxidase-conjugated anticytokeratin antibody and counterstain with haematoxylin (courtesy of Dr. E. Corsini, Fondazione IRCCS Istituto Neurologico Besta, Milano).

Figure 3 shows her CSF cytology with a representative cytokeratin-positive tumor cell.

This case underscores the heterogeneity of clinical course in neoplastic meningitis, since it conflicts with 2 other cases (both from a primary breast cancer) who are still alive at the present followup. Differences in the molecular biology profile of tumors within the same histotype are well known and may indeed play a role also in the more aggressive or indolent course of neoplastic meningitis. Note that in this case series the majority of patients did not present meningeal irritation signs/symptoms at disease onset.

When considering the toxicity profile, only one grade 4 toxicity occurred. In a melanoma patient, an inflammatory encephalopathy picture with seizures, stupor, signs of meningeal irritation, nausea, moderate increase in temperature took place starting 24 hours after intraventricular administration of 50 mg of liposomal AraC; concomitantly, a slight intraventricular CSF lymphocytosis was detected. The encephalopathy improved progressively leading to recovery of the premorbid status within 72 hours. CSF culture was negative for infectious complications.

4 more patients displayed moderate postinjection headache and slight fever, usually starting within 24 hours from intrathecal delivery of liposomal AraC and receding in 1 to 2 days.

2 patients—both affected by metastatic breast cancer—are alive at a followup ranging from 11 to 23 months.

## 5. Future Developments

Intrathecal chemotherapy for neoplastic meningitis may be a worthwhile option for a number of patients with this very serious disease. Technological developments allowing slow-release delivery of potentially active drugs may in the future be combined with targeted treatments (monoclonal antibodies, small molecule inhibitors) focused on multistep inhibition of neoplastic cell survival, growth, and spreading within the neuraxis.

However, a better basic knowledge of the biological mechanisms underlying selective homing of neoplastic cells to the leptomeninges, together with strict monitoring of the risk/benefit ratio [20, 21], will be needed before routine adoption of these approaches becomes a standard of care.

This is very important, since increased survival times are (also) the consequence of more aggressive systemic treatments, which may significantly enhance the neurotoxicity of intrathecal therapies [22–24].

## Figures and Tables

**Table 1:** Demographic features, site of primary tumor and PS.

Extra CNS tumor	26
Breast	13
Lung	7* (*1 pt lung and colon tumor)
Digestive system	3*
Melanoma	2
Unknown	1
Median age (range)	53 yrs (30–82)
Median KPS (range)	60 (20–100)

**Table 2:** Clinical signs and symptoms at onset of neoplastic meningitis.

Signs and symptoms and PS in extra CNS tumors	
Spinal cord and root symptoms and signs	9/26
Headache, Mental status change	6/26
Meningeal signs and headache	6/26
Cranial nerve symptoms and signs	4/26
Seizures	2/26

**Table 3:** Therapeutic management in the 26 patients of the cohort.

Control at primary site of disease	16 yes 10 no
Steroids	22/26
Radiotherapy	0/26
Systemic Chemotherapy	2/26
Intrathecal Depocyte	11/26 ( median 3 injections)
